# Correlation between Findings in Physical Examination, Magnetic Resonance Imaging, and Nerve Conduction Studies in Lumbosacral Radiculopathy Caused by Lumbar Intervertebral Disc Herniation

**DOI:** 10.1155/2020/9719813

**Published:** 2020-01-24

**Authors:** Safa Yousif, Afraa Musa, Ammar Ahmed, Ahmed Abdelhai

**Affiliations:** ^1^Department of Physiology, Faculty of Medicine, University of Khartoum, Khartoum, Sudan; ^2^Department of Orthopaedics Surgery and Traumatology, Faculty of Medicine, University of Khartoum, Khartoum, Sudan

## Abstract

**Purpose:**

The aim of this study was to find out the correlation between magnetic resonance imaging (MRI) and nerve conduction studies' (NCS) findings in patients with lumbosacral radiculopathy caused by lumbar intervertebral disc herniation. In addition, the study aimed at finding the correlation between the clinical manifestations of lumbosacral radiculopathy and both MRI and NCS. *Patients and Methods*. The study was a cross-sectional analytic study which included thirty patients with a history suggestive of lumbosacral radiculopathy. Inclusion criteria were as follows: patients who had an MRI confirmed L4/5 and/or L5/S1 intervertebral disc prolapse in addition to one or more of the following (dermatomal distribution of symptoms appropriate with MRI level, presence of motor weakness, sensory impairment, absent ankle jerk, or positive straight leg raising test). All patients underwent clinical assessment and NCS, and their MRI examination was reviewed. The Chi-Squared/Fisher's exact test was used to test the correlation.

**Results:**

There was a statistically significant correlation between abnormal physical findings and nerve root compression in MRI. Statistically significant correlation was neither found between abnormal physical examination findings and abnormal NCS nor between nerve root compression in MRI and abnormal NCS findings.

**Conclusion:**

Abnormal neurological examination findings can be used to predict nerve root compression in MRI examination. On the contrary, positive findings of physical examination do not predict abnormal NCS, as well as negative findings do not exclude abnormal NCS; therefore, it is useful to add NCS when MRI findings do not match clinical examination findings or when no neuroimaging abnormalities can be identified.

## 1. Introduction

Lumbar disc herniation is a common cause of low back pain in the third and fourth decade of life. Usually, pain is brought on by repetitive twisting, bending, or heavy lifting, and it starts in the lower back and radiates to the buttock and posterior thigh. The radicular pain usually extends below the knee following the involved nerve root dermatome [1]. Lumbosacral radiculopathy due to disc herniation is defined as localized displacement of disc material beyond the normal margins of the intervertebral disc space resulting in pain, weakness, or numbness in a myotomal or dermatomal distribution [2]. Clinical manifestations of lumbosacral radiculopathy range from sensory symptoms (paresthesia) to motor weakness and sphincteric disturbance or even cauda equina syndrome.

Clinical assessment and imaging studies are used to evaluate the patient's symptoms, and magnetic resonance imaging (MRI), being the most diagnostic importance, is employed to identify the etiology and determine the level of the anatomical abnormality. The clinical significance of the MRI findings has been questioned, although being highly sensitive for identifying disc problems; not all of the identified lesions cause symptoms [3]. Neural foramen compromise and multiple disc lesions are the radiological findings that are most likely to cause clinical symptoms [4, 5]. In some patients, MRI findings do not coincide with clinical findings, or the lesion cannot be identified (e.g., far extraforaminal lesion); therefore, sometimes the need arises to use another test to reach the diagnosis.

Nerve conduction studies (NCS) are electrodiagnostic tests (EDX) used to evaluate peripheral nerve functions in patients with neuromuscular complaints and can be used to diagnose radiculopathy [6]. EDX are not regularly used in our practice to assess patients with clinical lumbosacral radiculopathy. When compared with MRI, nerve conduction studies assess the physiological integrity of the nerve function, while MRI identifies structural abnormalities. NCS cannot identify the exact etiology of impaired nerve root function (e.g., disc herniation and tumour), but in contrast to MRI, it can detect far extraforaminal lesions (e.g., piriform fossa syndrome). Anatomical abnormalities identified by MRI aids in surgical planning, while NCS can determine the severity of nerve root damage and can be used for postoperative follow-up [7].

We all agree that the clinical examination is the most important tool to assess the patients. Few studies were performed to test the correlation between clinical examination, nerve conduction studies, and magnetic resonance imaging findings in lumbosacral radiculopathy.

The objectives of our study were to find the correlation between magnetic resonance imaging findings and nerve conduction studies' findings and between each of the two tests (MRI and NCS) and the physical examination findings in patients with lumbosacral radiculopathy caused by lumbar intervertebral disc herniation.

## 2. Materials and Methods

### 2.1. Study Design and Study Sample

This was a cross-sectional analytic study. Sample size was calculated by a statistician using Chi-squared equation, and the results of study were conducted by Soltani et al. in 2014 [8]. Sample technique used was a convenient sample. Two study groups were included: patients and control group. Subjects of the control group were selected from volunteers among workers and students of Faculty of Medicine of Khartoum University. Patients were selected from referred clinics (Ibrahim Malik Teaching Hospital-referred clinic and Ribat University Hospital-referred clinic) and specialized clinic (Mr. Ahmed Abdelhai clinic in Royal Scan diagnostic center) in Khartoum city.

### 2.2. Participants

#### 2.2.1. Patients Group

Thirty patients presented with a history suggestive of lumbosacral radiculopathy were included.

#### 2.2.2. Inclusion Criteria

(1) Patients who had an MRI confirmed L4/5 and/or L5/S1 intervertebral disc prolapse
(2) In addition to one or more of the following:
  - Dermatomal distribution of symptoms appropriate with MRI level
  - Presence of motor weakness
  - Sensory impairment
  - Absent ankle jerk
  - Positive straight leg raising test.

#### 2.2.3. Exclusion Criteria

1. A history suggestive of polyneuropathy (e.g., diabetes)
2. Radiculopathy due to other pathology (e.g., tumour and instability)
3. Previous spinal surgery

#### 2.2.4. Control Group

In order to obtain reference values for the NCS parameters, a control group was included and composed of twenty-five healthy volunteers with no history of radiculopathy, neuromuscular problems, or risk factors for having impaired nerve function besides normal neurological examination.

### 2.3. Data Collection Tools

Data collection was carried out from July to November, 2016. All patients underwent clinical assessment; their MRI films and reports were reviewed, and nerve conduction studies were performed at the Physiology department in the Faculty of Medicine of Khartoum University.

Clinical assessment included history taking and examination carried out by the principal investigator. The history included duration of symptoms, side affected, dermatomal distribution of symptoms, history of weakness or sphincteric disturbance, and previously received treatment. Symptoms considered to arise from L5 nerve root (L5 dermatomal distribution), if pain or numbness described by the patient radiates to the anterolateral leg, dorsum of the foot, or the great toe. S1 nerve root involvement is considered (S1 dermatomal distribution), if pain or numbness described by the patient radiates to the lateral malleolus, lateral, and planter surface of the foot or the heel. Physical examination included observation of the patient's gait, examination of lower limb tone and power, ankle jerk, pin prick sensation, and straight leg raising test.

All patients' MRI lumbosacral spine films (sagittal and axial T1- and T2-weighted sequences) were reported by a radiologist blinded to physical examination findings. MRI reports were used by the principal investigator to obtain the following information: the level of the involved intervertebral discs, extent, and localization of the disc prolapse, presence of nerve root compression, and neural canal compromise. Demographic data, history, clinical examination, and MRI findings were entered into special questionnaire.

Nerve conduction studies were performed by the principal investigator using the Digital Medelec Synergy machine and reviewed by a neurophysiologist blinded to the physical examination findings. Both lower limbs in all subjects were examined, and the tests performed include motor nerve conduction study of the tibial and common peroneal nerves. Tibial nerve compound motor action potential (CMAP) was recorded from abductor halluces muscle, two stimulation sites used: the distal one was posterior to the medial malleolus 10 cm proximal the recording electrode and a proximal stimulation site at the middle of the popliteal crease. Peroneal nerve CMAP was recorded from extensor digitorum brevis, and two stimulation sites were used: the distal one in dorsal lower leg between the tendons of tibialis anterior and extensor hallucis longus 9 cm proximal to active recording electrode and a proximal stimulation site about 3-4 cm distal to the proximal tip of fibular head. F-wave latencies of both nerves were recorded, using electrodes placement similar to that of motor studies. Ten subsequent stimuli were applied to the distal stimulation site, and F-waves recorded for latency measurements were the F-minimum, F-maximum, and F-mean latencies. H-reflex latency of the tibial nerve was recorded, posterior tibial nerve was stimulated in the centre of the popliteal crease and recorded over the soleus muscle, and the shortest latency recorded was the parameter studied. In addition, sensory nerve conduction study of the sural nerve was carried out to exclude patients with polyneuropathy. Machine setting for each test is illustrated in Table 1.

### 2.4. Analysis

Data analysis was carried out using Statistical Package for the Social Sciences (IBM SPSS) version 20 software. Descriptive statistics presented as mean and standard deviation for continuous variables and frequencies for categorical variables. Association between physical examination findings, NCS findings, and MRI findings were examined by Chi-Squared/Fisher's exact test. *P* value ˂0.05 was considered significant. Nerve root compression on MRI was considered as radiological evidence of radiculopathy. Reduced amplitude or conduction velocity of compound motor action potential, prolonged mean F-wave latency, and prolonged H-reflex latency were considered NCS findings suggestive of radiculopathy.

## 3. Results

The total number of patients included in the study was 30, with a mean age of 41.07 ± 11.47 years, 70% were males, and 30% were females. The number of healthy subjects was 25, with a mean age of 37.24 ± 10.4 years, 60% were males, and 40% were females.

### 3.1. Clinical Assessment

The mean duration of patients' symptoms was 21.47 ± 26 months, 60% of the patients have symptoms for more than 6 months. The left limb was involved in two-third (66.7%) of patients, right limb in 26.7%, while bilateral involvement occurred in 6.7% only. L5 dermatomal distribution of parasthesia was encountered in 40% of patients compared with 56.7% patients with S1 dermatomal distribution, while 3.3% only showed involvement of both dermatomes. Physical examination findings are demonstrated in Table 2.

### 3.2. MRI

MRI of 9 (30%) patients showed isolated L4/5 disc involvement, 7 (23.3%) showed isolated L5/S1 disc involvement, while 14 (46.7%) showed both L4/5 and L5/S1 disc level involvement. Distribution of the MRI findings among the patient group is shown in Table 3.

### 3.3. NCS

Concerning the compound motor action potential (CMAP) parameters, 6 (20%) patients showed reduced amplitude of the common peroneal nerve, and 3 (10%) patients showed reduced velocity. Reduced amplitude of the tibial nerve was detected in 2 (6.7%) patients, while 5 (16.7%) patients showed reduced velocity. Study of the F-wave of the common peroneal nerve showed prolonged mean latency of the symptomatic side in 6 (20%) patients. The mean latency of the F-wave of the tibial nerve was prolonged in 3 (10%) of the symptomatic sides. The most prevalent abnormality prolonged H-reflex latency which was detected in 12 (40%) of the studied patients.

### 3.4. Correlation Tests

There was statistically significant association between L5 dermatomal distribution of symptoms and L4/5 nerve root compression on MRI, and also statistically significant association was found between L5/S1 nerve root compression and S1 dermatomal distribution of symptoms (*P* < 0.05). The test of the correlation between abnormal physical findings and MRI detected nerve root compression revealed statistically significant association (*P* < 0.05) as presented in Table 4. Statistically significant association was neither found between abnormal NCS and abnormal physical examination nor between abnormal NCS findings and the presence of nerve root compression in MRI (*P* < 0.05) as shown in Tables 5 and 6, respectively. Sensitivity, specificity, and positive and negative predictive values of MRI and NCS in relation to physical examination findings are shown in Table 7.

## 4. Discussion

Magnetic resonance imaging is a sensitive tool in detecting structural lesion, while nerve conduction studies evaluate physiological changes in the nerve function. We conducted our study to find out the correlation between MRI findings and NCS findings in patients with clinical lumbosacral radiculopathy as well as their relation to the physical examination findings.

In our study, any degree of nerve root compression was considered as radiological findings of radiculopathy. There was no statistically significant association between abnormal physical examination findings (abnormal gait, absent ankle jerk, impaired sensation, and positive SLR test) and nerve root compression in MRI similar to previous studies [5, 8]. This would suggest that abnormal physical examination findings can predict nerve root compression in MRI. Soltani et al. conducted a study in 2014, and their results revealed a significant agreement between the clinical findings and relevant MRI; the results also showed that MRI had higher abnormalities than EDX in clinically irrelevant levels [8]. In our study, abnormal physical examination findings associated with the presence of nerve root compression is detected in 66.6% of patients (20 from total 30), compared with 71% and 58.6% in two previous studies [8, 9].

Prolonged H-reflex latency was the commonest encountered abnormality in NCS followed by prolonged F-wave latency. Other abnormalities detected include reduced amplitude and reduced conduction velocity. No statistically significant association was detected in the current study between physical examination and NCS findings; consequently, the physical examination is not a good predictor of abnormal nerve conduction studies. This agrees with the findings of many previous studies [10–13]. Our study results showed that 56.7% of patients had abnormal findings in physical examination as well as nerve conduction studies, compared with 58% and 89.5% in two previous studies [8, 9]. Electromyography (EMG) was used in conjunction with NCS to evaluate patients, and this might explain their higher figures. Lauder et al. concluded from their study that having at least one abnormal physical examination ﬁnding makes the probability of having an abnormal electrodiagnostic study more likely; however, having normal physical examination does not rule out the probability of finding abnormal EDX [11].

The current study failed to show a statistically significant correlation between MRI and NCS findings, and this corresponds with the findings of Soltani et al.'s study [8]. This can be explained by the fact that MRI concerned mainly with structural abnormalities, while NCS reflects the physiological integrity of the studied nerves [14]. Soltani et al. concluded from their study that MRI and EDX are complementary in general, and it is reasonable to add EDX when there is disagreement between MRI and clinical findings or when MRI neurologic findings are not detectable [8]. The current study showed that NCS has lower specificity compared with MRI in relation to the physical examination findings in contrast to previous studies [8, 9, 15]; this can be explained by inclusion of EMG in addition to NCS in the previous studies. MRI showed higher sensitivity compared with NCS, and this correlates with the findings of many previous studies [8, 9, 15]. The lower sensitivity and specificity of the NCS coincide with the findings of the correlation test (the presence or absence of positive findings in the physical examination does not predict positive or negative findings in the NCS). The lack of gold standard test to diagnose radiculopathy is one of limitation to the comparison between MRI and NCS diagnostic tests.

Our study supports the current practice of considering MRI as the gold standard investigation in diagnosing and detecting the cause of clinical radiculopathy, being very sensitive in detecting structural abnormalities and correlates with clinical findings. On the contrary, using NCS as an assessment tool has several limitations: one limitation is being not widely available in our settings and the other limitation is that specific root affected is difficult to be localized (each muscle supplied by several roots); adding EMG of lower limbs and paraspinal muscle would help to increase the specificity and determination of the specific root affected. Nevertheless, NCS has several advantages; the EDX is the only laboratory examination that directly assesses the physiologic integrity of the nerve root. In addition, EDX can identify changes when physical examinations are normal or inconclusive [16]. In certain cases, when neuroimaging studies cannot identify certain pathology, EDX may be abnormal (for example, patients with noncompressive radiculopathy) [16]. Another advantage is that imaging study cannot define the severity of axon loss; therefore, it cannot determine the prognosis in contrast to EDX [17]. Finally, nerve conduction studies are noninvasive tests and can be used to follow patients' progression over time or after operations [17, 18].

## 5. Conclusion

We can conclude from our study that abnormal neurological examination findings can be used to predict nerve root compression in MRI. On the contrary, positive findings of physical examination do not predict abnormal NCS, as well as negative findings do not exclude abnormal NCS. Furthermore, it is useful to add nerve conduction studies examination when clinical examination findings do not match MRI study findings or structural abnormalities cannot be identified by neuroimaging.

## Figures and Tables

**Table 1:** Machine setting for nerve conduction studies.

Setting	Sweep velocity (msec/division)	Sensitivity (*μ*v/division)	Filters high/low (kHz/Hz)	Stimulator duration/rate (msec/Hz)
Motor NCS	2	5000	10 kHz/2 Hz	0.2 msec/1 Hz
Sensory NCS	1	10	2 kHz/20 Hz	0.1 msec/2 Hz
H-reflex	10	500	10 kHz/20 Hz	1 msec/0.5 Hz
F-wave	10	500	10 kHz/20 Hz	0.2 msec/1 Hz

**Table 2:** Frequency of physical examination findings (number of patients: 30).

Positive sign	Number of cases
Antalgic gait	8 (26.7%)
Listing gait	7 (23.3%)
Weakness	0
Ankle jerk	9 (30%)
Impaired sensation	16 (53.3%)
Positive straight leg raising test	18 (60%)

**Table 3:** Frequency of MRI findings (number of patients: 30).

	L4/5 Number of levels = 23	L5/S1 Number of levels = 21
Axial localization *n* (%)	Diffuse: 9 (39.1%) Central disc: 7 (30.4%) Paracentral disc: 7 (30.40%)	Diffuse: 7 (33.3%) Central disc: 2 (9.5%) Paracentral: 12 (57.1%)

Extent *n* (%)	Herniation: 15 (65.2%) Migration: 1 (4.3%) Bulge: 7 (30.4%)	Herniation: 14 (66.7%) Migration: 2 (9.5%) Bulge: 5 (23.8%)

Nerve root compression *n* (%)	Left side: 9 (39.1%) Right side: 3 (13.04%) Bilateral: 3 (13.04%)	Left side: 7 (33.3%) Right side: 1 (4.7%) Bilateral: 5 (23.8%)

Central canal stenosis *n* (%)	10 (43.4%)	4 (19%)

Ligamentum flavum hypertrophy/facet joint arthropathy *n* (%)	2 (8.7%)	2 (9.5%)

**Table 4:** Association between abnormal physical examination and nerve root compression in MRI.

Physical examination	Nerve root compression in MRI			
	No compression	Compression	Total	*P* value
Normal	4 (13.3%)^*∗*^	3 (10%)^*∗*^	7	0.033
Abnormal	3 (10%)^*∗*^	20 (66.7%)^*∗*^	23	
Total	7	23	30	

^*∗*^Percent calculated from the total number of patients.

**Table 5:** Association between abnormal physical examination and abnormal NCS.

Physical examination	NCS			
	Normal	Abnormal	Total	*P* value
Normal	2 (6.7%)^*∗*^	5 (16.7%)^*∗*^	7	1.00
Abnormal	6 (20%)^*∗*^	17 (56.7%)^*∗*^	23	
Total	8	22	30	

^*∗*^Percent calculated from the total number of patients.

**Table 6:** Association between nerve root compression in MRI and abnormal NCSs.

Nerve root compression in MRI	NCSs			
	Normal	Abnormal	Total	*P* value
No compression	1 (3.3%)^*∗*^	6 (20%)^*∗*^	7	0.638
Compression	7 (23.3%)^*∗*^	16 (53.3%)^*∗*^	23	
Total	8	22	30	

^*∗*^Percent calculated from the total number of patients.

**Table 7:** Sensitivity, specificity, and positive and negative predictive values of MRI and NCS in relation to physical examination findings.

Study	Sensitivity (%)	Specificity (%)	Positive predictive value (%)	Negative predictive value (%)
MRI	87.0	57.1	86.9	57.1
NCS	65.2	28.6	75	20

## Data Availability

The data used to support the findings of this study are available from the corresponding author upon request.

## Supplementary Materials

Supplementary Materials Demographic data of the patient group, history, physical examination, MRI findings, and interpretation of NCS findings.
